# Plant Hormone Stimulation and *HbHSP90.3* Plays a Vital Role in Water Deficit of Rubber Tree (*Hevea brasiliensis* Muell. Arg.)

**DOI:** 10.3390/plants14233679

**Published:** 2025-12-03

**Authors:** Mingyang Liu, Songle Fan, Cuicui Wang, Bingbing Guo, Hong Yang, Phearun Phen, Lifeng Wang

**Affiliations:** 1 Key Laboratory of Biology and Genetic Resources of Rubber Tree, Ministry of Agriculture and Rural Affairs/State Key Laboratory Incubation Base for Cultivation & Physiology of Tropical Crops/Rubber Research Institute, Chinese Academy of Tropical Agricultural Sciences, Haikou 571101, China; liumy2023@126.com (M.L.); slfan@catas.cn (S.F.); wangcui915128@163.com (C.W.); guobingbing1989@126.com (B.G.); yang_hong0317@126.com (H.Y.); 2 Cambodian Rubber Research Institute, #59E, Street Betong, Phum Prek Leab, Sangkat Prek Leab, Khan Chroy Changva, Phnom Penh P.O. Box 1337, Cambodia; phearun_phen@yahoo.com

**Keywords:** *HbHSP90.3*, HbSGT1b, rubber tree, water deficit, phytohormone

## Abstract

The yield and quality of rubber tree latex are affected by environmental stresses and plant hormone stimulation. Heat shock protein 90 (HSP90) is widely involved in various developmental processes and stress responses in plants, especially in drought stress. In this study, we cloned the *HbHSP90.3* gene and characterized its expression pattern in different tissues and mechanical wounding treatments of the rubber tree and found that it is highly expressed in latex and responds to mechanical wounding treatment. To reveal the roles of plant hormones and HSP90.3 protein in the drought resistance process of rubber trees. Treatment with the specific HSP90 protein inhibitor geldanamycin (GDA) and yeast expression experiments demonstrated that *HbHSP90.3* has a relieving effect on water deficit in rubber trees. The expression pattern showed that the *HbHSP90.3* gene was closely related to hormone signaling, especially for Indole acid (IAA) and Zeatin (ZT) induction under different plant hormone treatments. Protein interaction analysis showed that HbHSP90.3 interacted with the suppressor of the G2 allele of skp1 (HbSGT1b). Taken together, HbHSP90.3 interacts with HbSGT1b in the nucleus and plays a key role in water deficit.

## 1. Introduction

The rubber tree (*Hevea brasiliensis* Muell. Arg.) is a major source of natural rubber and has significant socio-economic value in tropical regions around the world, e.g., China and Cambodia [1]. During their economic life cycle of 25–30 years, rubber trees are exposed to several abiotic stresses, such as seasonal drought [2], low-temperature chilling [3,4], and soil salinization, which significantly reduce the latex yield and quality through mechanisms that inhibit latex laticifer differentiation and impede natural rubber biosynthesis and latex flow. Rubber tree genomic data has shown that rubber trees are different from many other rubber-producing plants, arguably because of rubber elongation factor (REF), small rubber particle protein (SRPP), and cis-Prenyltransferases (CPTs), which are actively involved in natural rubber biosynthesis to increase rubber production [5]. Previous studies have shown that drought stress adaptation in rubber trees is related to energy biosynthesis, antioxidative enzymes, and osmoregulation [2]. Water and plant hormones play important roles in the growth and development, laticifer differentiation, and latex flow of rubber trees [6]. Plant hormones are involved in the production and flow of latex within the rubber tree as regulators [7]. Stimulation of ethephon (ETH) and methyl jasmonate (MeJA) causes significant physiological and metabolic changes in latex cells. Ethephon can increase the yield of rubber trees by prolonging the flow period of latex and accelerating latex regeneration and is widely used as a yield enhancer in production [8]. Methyl jasmonate can promote the differentiation of secondary milk ducts in rubber trees and upregulate the expression of genes related to rubber biosynthesis to regulate the biosynthesis of natural rubber [9]. Brassinolide (BR) is an important plant hormone that regulates plant growth, development, and yield. BR improves latex yield and quality by reducing the clotting index and increasing the emulsion rupture index without reducing mercaptans, sucrose, and inorganic phosphorus [10]. Auxin and gibberellin (GA) play an important role in almost all aspects of the plant life cycle and have a mitigating effect on the occurrence of dead bark in rubber trees. Gibberellin plays an important regulatory role in seed germination, stem elongation, leaf development, and flower transformation [11]. Auxin is polarly distributed in plant tissues and organs, with similar morphogenetic activity during plant development. However, little is known about the inner molecular mechanisms for drought stresses in rubber trees.

Heat shock protein 90 (HSP90), an important class of molecular chaperone proteins, regulates the growth and development of animal/plant cells and the innate immune response due to its effect on the conformation of various client proteins [12]. Accordingly, HSP90 can also be involved in plant growth and development under normal and stress conditions by regulating cellular protein homeostasis [13]. In *Arabidopsis*, overexpression of *AtHSP90.2*, *AtHSP90.5*, and *AtHSP90.7* enhances plant sensitivity to salt and drought stress [14]. *OsHSP90.2* significantly improves resistance to various stresses in rice, such as heat, high salinity, and drought [15]. Transcriptional analysis of the *BdHSP90* genes under salt and drought stress conditions indicated that the expression of these genes was delayed or increased at different stress time points [16]. HSP90 refolds damaged proteins when the plant is subjected to heat stress, enabling plants to become heat-tolerant [17]. Proteomic analysis showed that the accumulation of HSP90 in *Alfalfa* increased after heat stress treatment [18]. The seedling root growth activity of mutant *Athsp90-1* decreased more significantly than wild-type (WT) *Athsp90-1* under water deficit conditions [19]. Outside of mutant analysis, geldanamycin (GDA) and radicicol (RAD) have been shown to competitively bind to the ATP-binding pocket with high affinity, thereby serving as specific HSP90 ATPase inhibitors that have also been widely used to reveal the function of the HSP90 protein in plants [20]. Exogenous application of GDA results in a variety of developmental phenotypes, such as growth hindrance, curly leaves, and hypocotyls [21,22]. Inhibition of HSP90 by exogenous GDA use in *Arabidopsis* affects embryo development and the pattern formation of veins in cotyledons [23]. After GDA inhibits HSP90, ATPase activity causes hyperphosphorylation of the transcription factor BRI1-EMS SUPPRESSOR 1 (BES1) and disrupts the expression of Brassinolide (BR)-responsive genes, suggesting that HSP90 is necessary for maintaining BES1 dephosphorylation status in the BR signaling pathway [24]. HSP90 functions as a specialized chaperone protein binding to client proteins. In Cassava, MeHSP90.9 interacts with the transcription factor MeWRKY20 and the catalase MeCatalase1 to regulate abscisic acid (ABA) synthesis and reactive oxygen species (ROS) scavenging, thereby enhancing drought tolerance. Chloroplast-targeted HSP90.5 interacts with vesicle-inducing protein in plastids 1 (VIPP1) and has an essential function in plastid development and embryogenesis in *Arabidopsis* [25]. The HSP90-YODA module regulates phosphorylation-dependent inactivation of SPEECHLESS (SPCH) to control stomatal development under acute heat stress [23]. Moreover, HSP90, along with the co-chaperone suppressor of the G2 allele of skp1 (SGT1), has been widely shown to be involved in the regulation of plant stress responses [26]. SGT1 is a conserved eukaryotic protein with two family members in rubber tree: SGT1a and SGT1b, both of which play crucial roles in rubber tree growth and development, as well as in regulating stress responses [27]. In *Arabidopsis*, SGT1b regulates auxin responses via the SCF^TIR1^ complex. *SGT1b* overexpression partially counteracts the auxin response defect conferred by the *tir1-1* mutation [28]. The plant root system requires the HSP90-SGT1 chaperone system to cope with elevated ambient temperatures and functions by stabilizing the growth hormone co-receptor F-box protein TIR1 [29]. HSP90 and SGT1 interaction regulate plant disease resistance. Virus-induced gene silencing (VIGS) targeting HSP90 and SGT1 in tomato showed enhanced susceptibility to tomato chlorosis virus (ToCV) [30]. These results suggest that HSP90 is essential for plant growth and development, as well as stress responses.

Although HSP90 has been extensively studied in other plants, its regulatory mechanism in rubber tree drought stress has not been elucidated. We identified seven HbHSP90 proteins in rubber tree and classified them into three groups [31]. Based on these results, we propose the hypothesis that HbHSP90 is involved in the mechanism of rubber tree water deficit regulation. This study confirmed the role of *HbHSP90.3* in water deficit and plant hormone stimulation in the rubber tree. In addition, the effects of HbHSP90.3 and the chaperone HbSGT1b were also revealed. This study provides key theoretical support for elucidating the molecular mechanism by which *HbHSP90.3* regulates the drought stress of rubber tree.

## 2. Results

### 2.1. Cloning and Expression Analysis of HbHSP90.3

**Figure 1:**
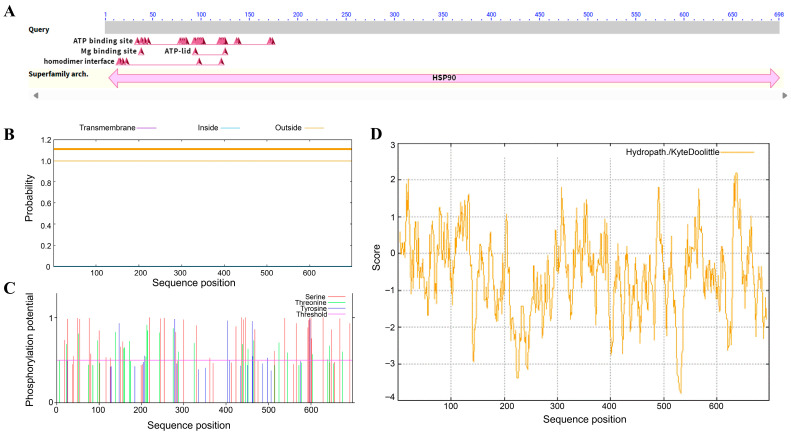
Bioinformatics analysis of HbHSP90.3 protein of rubber tree. (**A**) Conserved domain of HSP90 superfamily. (**B**) Transmembrane analysis of HbHSP90.3 protein. (**C**) Phosphorylation sites of HbHSP90.3 protein. (**D**) Hydrophilic analysis of HbHSP90.3 protein.

**Figure 2:**
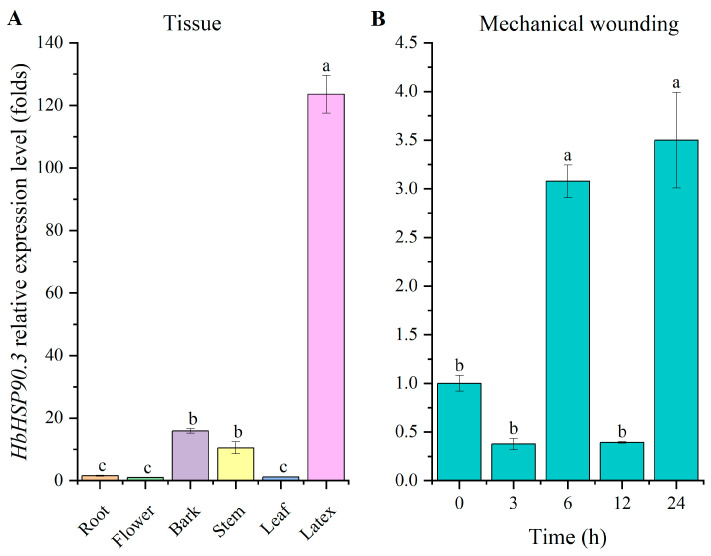
Expression patterns of the *HbHSP90.3* gene in different tissues of rubber tree (**A**) and after mechanical wounding treatments (**B**). The error bars represent the standard deviation of three independent experiments. Different lowercase letters are used to indicate significant differences at the *p* < 0.05 level.

The *HbHSP90.3* gene was cloned from a leaf of the rubber variety ‘CATAS73397’. The total length of this gene was 2179 bp, with a 2097 bp coding region and 698 amino acids (GenBank number: OP375587.1). The protein conserved domain analysis results indicate that HbHSP90.3 has a conserved HSP90 domain (Figure 1A). The protein characterization results indicate that it is hydrophobic and does not contain transmembrane domains (Figure 1B,D). It primarily contains phosphorylation sites at serine, threonine, and tyrosine (Figure 1C). The expression patterns of *HbHSP90.3* genes in different tissues of rubber trees were analyzed using RT-qPCR (Figure 2). The results show that *HbHSP90.3* was differentially expressed in the root, flower, bark, stem, leaf, and latex of rubber trees, but the expression was higher in latex. The *HbHSP90.3* expression pattern in response to mechanical wounding shows that *HbHSP90.3* was significantly upregulated at 6 and 24 h.

### 2.2. HbHSP90.3 Relieves Rubber Tree Water Deficit

**Figure 3:**
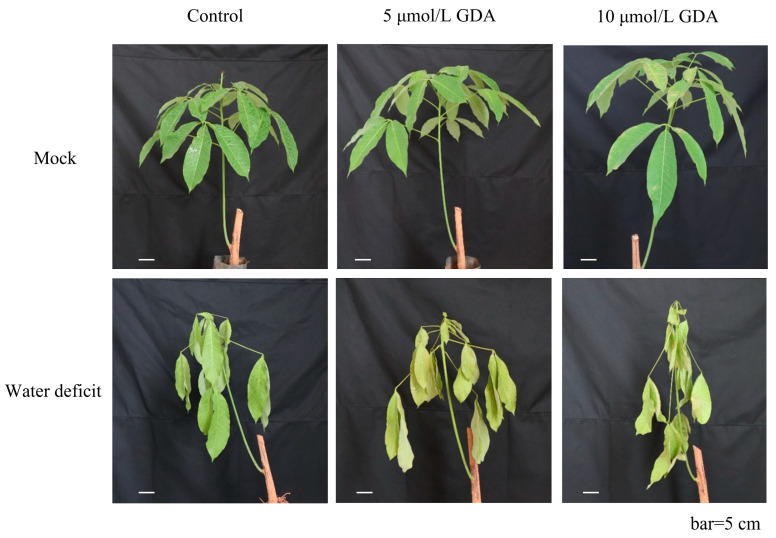
Effects of GDA on water deficit in rubber trees grafted seedlings. Phenotypes of control, 5 μmol/L GDA-, and 10 μmol/L GDA-treated plants after 6 d. Bar = 5 cm.

A limited supply of water can affect the growth and development of plants and severely limit their production [32]. Gerdermycin (GDA), which is a specific HSP90 protein inhibitor. By inhibiting the function of HbHSP90 protein and observing the response of rubber trees to drought, the function of HbHSP90 protein under normal conditions can be inferred backwards. Under water deficit conditions, GDA-treated rubber tree leaves showed a more severe dehydration phenotype than the control plants, and the 10 μmol/L GDA-treated plants showed more obvious wilting symptoms compared with the 5 μmol/L GDA-treated group (Figure 3). Under the same drought conditions, rubber trees treated with GDA (i.e., HbHSP90 function was partially inhibited) showed more severe dehydration and wilting than the untreated control group. This suggests that normal HbHSP90 protein is necessary to help plants resist drought.

**Figure 4:**
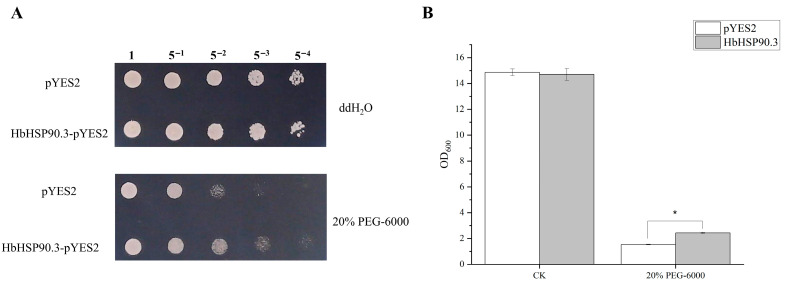
*HbHSP90.3* relieves water deficit in yeast. (**A**) Yeast treated with PEG-6000. (**B**) Yeast growth under 20% PEG-6000 treatment. Error bars indicate the standard deviation (SD) of three independent experiments. An asterisk (*) indicates a significant difference at the *p* < 0.05 level.

To further verify the regulatory effect of *HbHSP90.3* on water deficit in rubber trees, we constructed the recombinant yeast HbHSP90.3-pYES2 and the control yeast pYES2 under 20% PEG-6000 treatments. The pYES2 vector is routinely employed in yeast stress-resistance gene screening experiments. Specifically designed to monitor the inducible expression of recombinant proteins in yeast, this vector features the GAL inducible promoter system. It enables robust induction of heterologous protein expression in the presence of galactose, while suppressing such expression under glucose conditions. Additionally, the incorporation of the URA3 selectable marker permits effective screening on synthetic dropout media lacking uracil (SD-URA). The results show that there was no significant difference in the growth status between HbHSP90.3-pYES2 and pYES2 in the no-stress treatments. However, HbHSP90.3-pYES2 showed significantly more plaque growth than pYES2 under the 20% PEG-6000 treatment (Figure 4A). In addition, the growth differences between the recombinant yeast HbHSP90.3-pYES2 and control yeast pYES2 under liquid-induced culture conditions at 36 h were also compared. The results show that there was a significant difference between yeast that contained HbHSP90.3-pYES2 and pYES2 under the 20% PEG-6000 treatment (Figure 4B). This is a very classic method of verifying gene function. Under normal conditions, there was no difference between yeast containing HbHSP90.3 gene and empty carrier yeast, indicating that the gene itself had no negative effect on normal growth. However, under drought stress simulated by 20% PEG-6000, yeast colonies expressing HbHSP90.3 were larger and more vigorous in liquid medium. This result directly proves that the introduction of HbHSP90.3 gene alone is enough to give yeast cells stronger drought resistance. This strongly indicates that the protein encoded by this gene itself has the function of protecting cells and mitigating water stress damage. These results suggest that the *HbHSP90.3* gene is required to alleviate water deficit.

### 2.3. HbHSP90.3 Gene Responds to Plant Hormone Treatments

**Figure 5:**
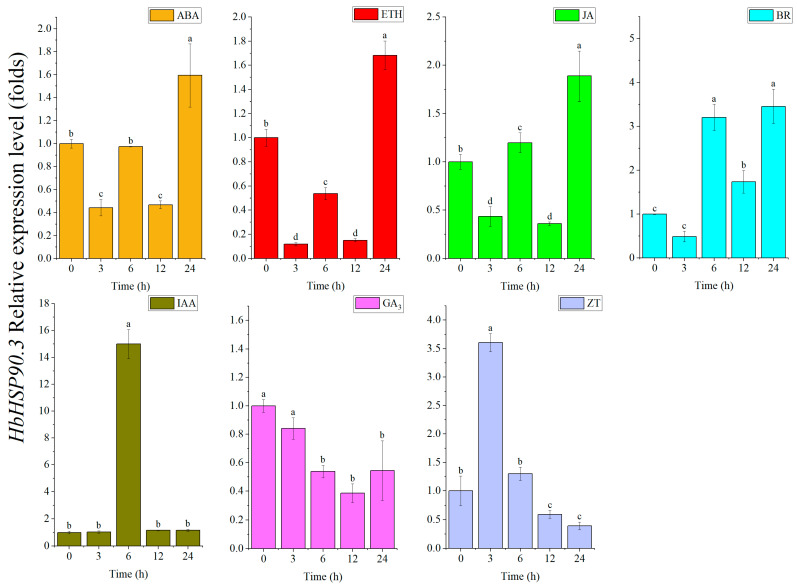
*HbHSP90.3* expression patterns in rubber tree leaf treated with plant hormones. Error bars represent the standard deviation of three independent experiments. Different lowercase letters are used to indicate significant differences at the *p* < 0.05 level by analysis of variance.

Plant hormones play a crucial role in helping plants adapt to adverse environmental conditions. To explore the *HbHSP90.3* gene’s response in rubber tree after the plant hormone treatment, we analyzed the *HbHSP90.3* gene’s expression pattern using RT-qPCR under different plant hormone treatments in rubber tree leaves (Figure 5). The results show that *HbHSP90.3* expression was upregulated in leaves under ABA, ethephon (ETH), methyl jasmonate (MeJA), and brassinolide (BR) treatments, and peaked at 24 h after treatment. Under zeatin (ZT) and indoleacetic acid (IAA) treatments, the *HbHSP90.3* gene expression was first upregulated and then downregulated, and the expression levels were significantly upregulated at 3 and 6 h. However, the *HbHSP90.3* gene expression was downregulated with gibberellin (GA_3_) treatment. These results suggest that the *HbHSP90.3* gene was significantly affected by the hormone treatments.

### 2.4. HbHSP90.3 and HbSGT1b Interacted in Tobacco Nucleus

**Figure 6:**
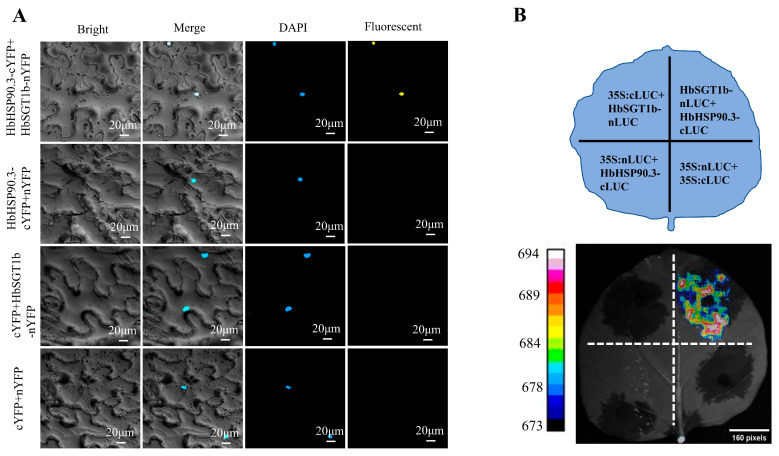
HbHSP90.3 interacts with HbSGT1b in tobacco (*Nicotiana benthamiana*) leaf. (**A**) BiFC assay validates protein interaction. The HbHSP90.3-cYFP and HbSGT1b-nYFP interaction results in a YFP fluorescent signal. Bar: 20 μm. (**B**) LCA validate protein interactions. Interaction between HbHSP90.3-cLUC and HbSGT1b-nLUC generates a fluorescent complex in tobacco (*Nicotiana benthamiana*) leaf. In contrast, there is no fluorescence when control vectors are injected into tobacco (*Nicotiana benthamiana*) leaf.

To understand the molecular chaperone function of HbHSP90.3 in rubber tree, we investigated the binding activity of HbHSP90.3 to HbSGT1b. We demonstrated that HbHSP90.3 interacted with HbSGT1b using bimolecular fluorescence complementation (BiFC) (Figure 6A). The HbHSP90.1-cYFP and HbSGT1b-nYFP vectors were constructed to transform Agro-bacterium GV3101 and injecting into tobacco leaves. It can be found that only cYFP+nYFP, cYFP+ HbSGT1b-nYFP, HbHSP90.1-cYFP+nYFP cannot showed fluorescent in nucleus, only HbHSP90.1-cYFP and HbSGT1b-nYFP can interact and show fluorescent in nucleus. Furthermore, the interaction between HbHSP90.3 and HbSGT1b was further shown using a luciferase complementation assay (LCA) (Figure 6B). Both experiments showed that HbHSP90.3 can interact with HbSGT1b.

## 3. Discussion

Natural rubber (*cis*-1, 4-polyisoprene) makes up about one-third of the volume of latex, which is essentially the cytoplasm of the articulated laticifers in rubber tree. Other than the general cytoplasm, it contains rubber particles, lutoid, Frey–Wyssling complex, and other unique organelles. Rubber trees cannot be replaced by industrial synthetic rubber in some areas because of their unique physical properties [33]. Rubber trees face various stresses under growth conditions, which leads to the decline in yield and quality of latex. With climate change, drought has also become a major disaster affecting rubber plantations in China and Cambodia. The role of *HSP90* gene regulation in plant responses to drought stress has been extensively studied, but research in rubber trees is scarce. This study cloned and analyzed the protein characteristics of the *HbHSP90.3* gene (Figure 1). The results show that the HbHSP90.3 protein primarily contains phosphorylation sites at serine, threonine, and tyrosine residues, which significantly influence its regulation of ATPase activity and binding to client proteins. Natural rubber biosynthesis pathways involve key enzymes, such as rubber transferase, which resides within the core enzyme complex on the rubber particle membrane. This enzyme catalyzes the polymerization of isopentenyl pyrophosphate (IPP) into rubber particles [34]. The complex itself is unstable and requires the assistance of HSP90 to fold and maintain activity correctly. We analyzed *HbHSP90.3* expression pattern in different rubber tree tissues. It was found that *HbHSP90.3* was significantly expressed in latex (Figure 2A). Thus, we hypothesized that HbHSP90.3 may participate in protein assembly, transport, and degradation in the isoprene biosynthesis pathway. Latex is extracted by tapping the bark, a non-destructive method of harvesting that facilitates continual production. This process is similar to plant root exudation [35]. Each tap is a disturbance to the laticifer cells. We analyzed the *HbHSP90.3* expression pattern during mechanical wounding, and the results show that *HbHSP90.3* was upregulated (Figure 2B). We hypothesized that HbHSP90.3 participates in regulating the denaturation and inactivation of key proteins caused by mechanical and oxidative damage resulting from latex tapping to ensure that lactiferous cells can continue functioning normally under continuous tapping.

Drought is one of the primary abiotic stresses resulting from water deficit. Water achieves biomass accumulation under good conditions by favoring stomatal opening, organ growth, and plant metabolism. The first effect of water deficit in plants is a drastic reduction in the growth of expanding tissues, and the lack of water in the nutrient phase affects leaf growth and light interception [36]. Rubber trees contain an average of 60% to 70% water in latex; sufficient available water to approach maximum turgor pressure in the laticiferous cell is required for good expulsion of latex [37]. Hence, a water deficit is an important stress factor limiting the yield of rubber trees. Rubber trees under water deficit conditions reduce net CO_2_ assimilation, leaf area, chlorophyll synthesis, and total dry matter accumulation, which results in more dry, yellow, and fallen leaves, leading to lower latex yields [38]. Heat shock proteins are known to be expressed in response to a variety of abiotic stresses, including heat, cold, hypoxia, and ultraviolet light stresses [39]. Heat shock protein expression enhances water retention behavior in citrus under water deficit conditions [40]. In drought treatment, the expression of *RcHSP90* in rose showed a trend of decreasing, then increasing, then decreasing with the increase in stress time, indicating that *RcHSP90* plays a positive role in regulating drought in rose [41]. In this study, we determined that *HbHSP90.3* had an alleviating effect on water deficit in rubber trees using HSP90-specific protein inhibitor GDA and yeast expression experiments (Figure 3 and Figure 4), which is consistent with the results of previous studies [42].

Plants have evolved a sophisticated molecular mechanism to cope with water deficit, with plant hormones serving as core endogenous signaling molecules that play a crucial role in sensing, transmitting, and responding to drought signals [43,44]. ABA is a core regulator of the drought response. Upon detecting a water deficit, roots rapidly synthesize and transport ABA through the vascular system to the aboveground parts, triggering defensive responses, such as stomatal closure and reduced leaf expansion [45]. Ethylene (ET) shows a concentration-dependent dual effect in response to drought. Under mild drought conditions, low concentrations of ET and ABA synergistically promote stomatal closure. However, continued drought leads to substantial ethylene accumulation, which accelerates leaf senescence and flower abscission, thereby reducing growth rates and photosynthetic efficiency [46]. JA as a defense hormone exhibits significant synergistic effects with ABA. JA co-regulates stomatal closure and antioxidant gene expression with ABA signaling through the COI1-JAZ-MYC2 signaling module, thereby alleviating drought-induced oxidative damage [47]. BR is an important plant hormone that regulates plant growth and development. They alleviate stress-induced inhibition by promoting growth recovery, while also positively regulating drought tolerance through enhancing antioxidant defense systems and interacting with ABA signaling [44]. The spatial redistribution of auxin is a key strategy for drought adaptation. Drought induces changes in the phosphorylation status of PIN proteins, leading to IAA transport toward the root tip. This promotes primary root elongation and lateral root formation, optimizing the water absorption architecture. Meanwhile, the weakening of IAA signaling in the aboveground parts reduces stomatal opening and leaf expansion, thereby decreasing the transpiration area [48]. Altered GAs biosynthesis affected the levels of many amino acids in leaf and root tissues, mainly under water deficit stress [49]. These amino acids are mainly involved in regulating membrane permeability, stomatal opening (as an osmotic protective agent), detoxification, ion transport, redox homeostasis, and enzyme activity. ZT, a kind of cytokinin, is a new type of plant growth regulator that can stimulate plant cell division, accelerate plant metabolism and protein synthesis, and improve plant disease resistance and anti-aging ability [50]. In addition, it has been shown that HSP90 is involved in the regulation of several plant hormones [51]. HSP90 can show auxin responsive phenotypes in plants harboring a cryptic deleterious point mutation in the TIR1 gene [52]. HSP90.3 was shown to be required for maintaining the dephosphorylated state of the transcription factor BES1 in the BR signaling pathway [24]. JA response genes are differentially regulated when seedlings are treated with GDA inhibitors, suggesting that HSP90 is involved in the JA response [53]. In this study, *HbHSP90.3* was found to be induced and regulated by the plant hormones (Figure 5). We hypothesize that *HbHSP90.3* may play a crucial role in rubber tree water deficit responses by regulating plant hormone pathways.

**Figure 7:**
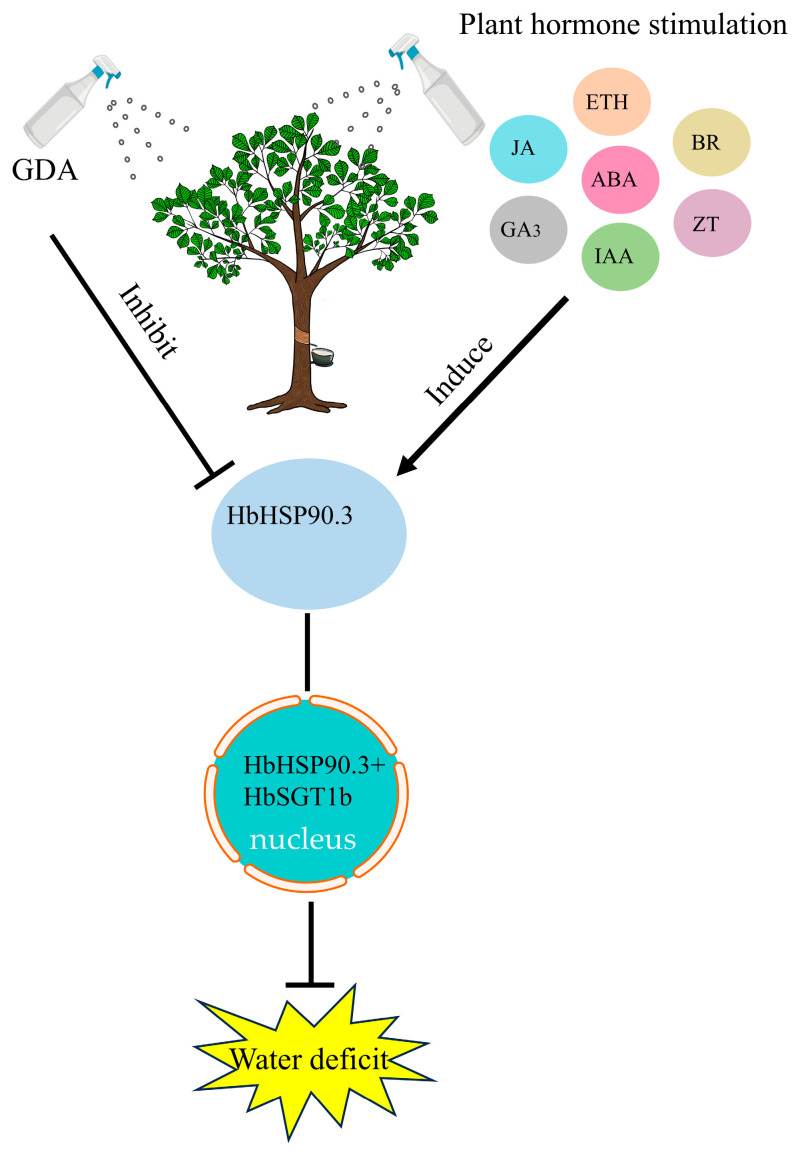
HbHSP90.3 interacts with HbSGT1b and plays key role in water deficit.

Heat shock protein HSP90 and its chaperone partner SGT1 form a highly conserved molecular chaperone system in plants responding to environmental stress. The interaction between HSP90 and SGT1 has been studied primarily in biotic stress conditions. For example, overexpression of *MeHSP90.9*, *MeSGT1*, and *MeRAR1* increase the resistance of Cassava leaves to bacterial blight, while *MeHSP90* silencing reduces cassava resistance [54]. Silencing of *Hsp90* decreases tomato resistance gene *Tm-2^2^*-mediated resistance to *Tobacco* mosaic virus (TMV) and homeostatic levels of the Tm-2^2^ protein. However, the Hsp90-SGT1 complex regulates Tm-2^2^ stability and is involved in Tm-2^2^-mediated TMV resistance [55]. Drought stress can cause protein refolding and aggregation within plant cells. HSP90-SGT1, as a molecular chaperone system, recognizes and binds to partially folded or misfolded proteins, promoting their proper folding or targeted degradation in an ATP-dependent manner [56]. In *Arachis*, genome-wide analysis identified 19 *HSP90* genes and 6 *SGT1* genes, several of which play key roles in salt and drought stress responses [57]. In this study, we demonstrated the interaction between HbHSP90.3 and HbSGT1b through BiFC and LCA (Figure 6). We speculate that HbHSP90.3 and HbSGT1b interact to maintain intracellular homeostasis, thereby regulating the water deficit in rubber tree (Figure 7).

## 4. Materials and Methods

### 4.1. Plant Materials and Treatments

The rubber tree variety ‘CATAS73397’ planted in the experimental field of the Rubber Research Institute of the Chinese Academy of Tropical Agricultural Sciences (CATAS) in Danzhou City, Hainan Province, China (19°51′51 N; 109°55′63 E), was selected as the experimental material for different tissue and hormone treatments. Different hormone treatments were prepared as follows: 1.5% (*v*/*v*) ETH, 200 μmol/L MeJA, 1 μmol/L BR, 3 mmol/LGA_3_, 66 μmol/L IAA, 2 mg/L ZT, and 200 μmol/L ABA. All samples were stored at −80 °C. All plant hormones were purchased from Merck Ltd., Beijing, China. These hormones were evenly applied to the tapping panel with a brush about 2 cm above and below the tapping line of the rubber tree. The mechanical wounding of rubber trees was performed by inserting a thumbtack every 5 cm along the tapping line, starting 3 cm below the tapping line. The thumbtacks remained in place until sampling was complete. Latex was collected at 0, 3, 6, 12, and 24 h for the treatment, and 0.05% (*V*/*V*) alcohol was used as the control. All were collected using liquid nitrogen. Budded seedlings of the rubber tree variety ‘CATAS73397’ grown in plastic pots in a chamber with vermiculite and turfy soil (1:3) were used in the rubber tree water deficit treatment. These seedlings were planted at an average temperature of 30 °C, precipitation of 180 mm, and humidity of 97.5% during the growing season, with a light intensity of 100 μmol m^−2^ s^−1^ [58]. The control group continued to be cultured under natural conditions. Concentrations of 5 and 10 μmol/L HSP90-specific protein inhibitor GDA were prepared and uniformly sprayed on the rubber leaves, and the same volume of Dimethyl sulfoxide (DMSO) was used as a negative control. The tobacco (*Nicotiana benthamiana*) seeds used for the transient gene expression were placed on a substrate with a 2:1 volume ratio of grass charcoal soil to vermiculite and incubated for germination. The culture conditions were 16 h/d at 26 °C in the light and 8 h/d at 23 °C in the dark, with a relative humidity of 70% and a light intensity of 100 μmol m^−2^ s^−1^.

### 4.2. Yeast Expression and Real-Time Quantitative PCR (RT-qPCR) Analysis

The CDS sequence of *HbHSP90.3* was amplified into the expression vector pYES2, which was sequenced and verified, and then transfected into the *Saccharomyces cerevisiae* strain INVSC1. The PCR that tested pYES2-HbHSP90.3 was cultured with MaxQ™ 8000 Incubated/Refrigerated Stackable Shakers (Thermo Fisher Scientific Inc., Carlsbad, CA, USA) to an OD_600_ = 1.0 measured with NanoDrop Ultra Microvolume UV–Vis Spectrophotometers (Thermo Fisher Scientific Inc., Carlsbad, CA, USA). The cultures were resuspended in control and 20% PEG-6000. For the solid selective medium induction, the cultures were spot plated after five gradient dilutions, and the growth differences were compared after 3 d of incubation. For the liquid medium induction, the cultures were adjusted with OD_600_ = 0.4, then 10 mL of the solution was taken separately and incubated up to 36 h to compare the growth differences between HbHSP90.3-pYES2 and the control yeast pYES2 [59].

The gene sequences of *HbHSP90.3* were obtained from the rubber tree genome, and RT-qPCR primers were designed based on the CDS region of the gene sequences. Construction of the expression vector primers was combined with expression vector mapping to design homologous recombination primers with insertion fragments for enzymatic sites. All primers were designed using The Vazyme Company’s website https://en.vazymemedical.com/ (accessed on 1 March 2024) (Table 1). RNA isolation, cDNA synthesis, and RT-qPCR were performed according to a previous study [60]. *HbACTIN* (HQ260674.1) was the reference gene, and the gene expression level was calculated using the 2^−^^ΔΔCT^ method [61]. The gene expression was normalized to control for the unstressed expression level, which was assigned a value of 1. Three biological repeats and three experimental replicates were carried out for each sample.

### 4.3. Luciferase Complementation Assay (LCA)

The CDS sequences of HbHSP90.3 and HbSGT1b were amplified into the expression vectors pCAMBIA1300-cLUC and pCAMBIA1300-nLUC, respectively. The vector construction and infestation processes were performed according to previous methods [31,62]. During observation, D-luciferin potassium salt was injected into each region on the dorsal side of the tobacco leaf. The leaf luminescence was detected using a live plant molecular imaging system (CDD imaging system, purchased from Guangzhou Kona Import & Export Ltd., Guangzhou, China), and the images were processed using Image J software version 1.54j [63].

### 4.4. Bimolecular Fluorescence Complementation (BiFC) Assay

The CDS sequences of HbHSP90.3 and HbSGT1b were amplified into the expression vectors pSPYCE and pSPYNE, respectively. The HbHSP90.1-cYFP and HbSGT1b-nYFP vectors were constructed to transform *Agrobacterium GV3101*. The bacterial solution was injected into five-week-old tobacco leaves and observed 30–48 h after injection. For observation, the leaf epidermis was torn and stained with 4′, 6-diamidino-2-phenylindole (DAPI) and washed three times with 0.9% saline. The treated tobacco leaves were placed under a laser confocal microscope with excitation light at a wavelength of 514 nm to observe the YFP fluorescence signal with LSM800 confocal microscopes (Carl Zeiss Industrielle Messtechnik GmbH, Oberkochen, Germany). Three biological replicates of tobacco were selected for the fluorescence detection.

### 4.5. Statistical Analysis

Data entry and calculations were conducted employing Excel 365 (Microsoft, Redmond, WA, USA). The AVERAGE and STDEV functions were used to calculate the mean and standard error of CP values. Variance analysis was executed utilizing SPSS 27 (IBM, Armonk, NY, USA). All graphical representations were generated with the aid of Origin 2021 software (OriginLab Corporation, Northampton, MA, USA).

## 5. Conclusions

The practice of tapping rubber trees results in significant water loss in the bark, leading to a water deficit and inducing physiological alterations, including the proliferation of reactive oxygen species (ROS). In this investigation, we isolated the *HbHSP90.3* gene and demonstrated that plant hormones and the HSP90.3 protein synergistically contribute to the tolerance of rubber trees to water deficit through the utilization of GDA inhibitors and protein interaction methodologies. Specifically, the administration of plant hormones increased the expression of the HSP90.3 protein, which engages in an interaction with HbSGT1b within the nucleus, thereby augmenting the drought resistance of rubber trees. In summation, the HbHSP90.3 protein plays a pivotal role in the water deficit response of rubber trees. The subsequent phase of research intends to utilize transgenic and RNAi technologies to thoroughly validate the structure and function of HbHSP90.3 from both forward and reverse genetic perspectives.

## Figures and Tables

**Table 1:** Primers used for *HbHSP90* gene RT-qPCR and vector construction. Note: The endonuclease sequence is underlined.

Primers	Function	Sequences 5′–3′
*HbHSP90.3-QF*	qRT-PCR	CTTGACCAACGACTGGGAGG
*HbHSP90.3-QR*		GCTCATTTTCTTGCGGGTGT
*HbACTIN-F*		GATGTGGATATCAGGAAGGA
*HbACTIN-R*		CATACTGCTTGGAGCAAGA
*HbHSP90.3-F*	Yeast expression	CTTGGTACCGAGCTCGGATCCATGGCTGATGCTGAGACCTTCG
*HbHSP90.3-R*		TAGATGCATGCTCGAGCGGCCGCTTAGTCGACTTCCTCCATCTTGC
*HbHSP90.3-F*	BiFC	TGGCGCGCCACTAGTGGATCCATGGCTGATGCTGAGACCTTCG
*HbHSP90.3-R*		GTACATCCCGGGAGCGGTACCGTCGACTTCCTCCATCTTGCTC
*HbSGT1b-F*		CCCAGGCCTACTAGTGGATCCATGGCGTCTGATCTCGAAAGG
*HbSGT1b-R*		CTCCTACCCGGGAGCGGTACCTCAATACTCCCATTTCTTCACCTCC
*HbHSP90.3-F*	LCA	TACGCGTCCCGGGGCGGTACCATGGCTGATGCTGAGACCTTCG
*HbHSP90.3-R*		TGTAGTCCATTTGTTGGATCCTTAGTCGACTTCCTCCATCTTGC
*HbSGT1b-F*		GTCGACGGTATCGATAAGCTTATGGCGTCTGATCTCGAAAGG
*HbSGT1b-R*		TTTACTCATACTAGTGGATCCATACTCCCATTTCTTCACCTCCAT

## Data Availability

All data generated or analyzed are included in the article.
